# SeeTB: A novel alternative to sputum smear microscopy to diagnose tuberculosis in high burden countries

**DOI:** 10.1038/s41598-019-52739-9

**Published:** 2019-11-12

**Authors:** Vikas Pandey, Pooja Singh, Saumya Singh, Naresh Arora, Neha Quadir, Saurabh Singh, Ayan Das, Mridu Dudeja, Prem Kapur, Nasreen Zafar Ehtesham, Ravikrishnan Elangovan, Seyed E. Hasnain

**Affiliations:** 1 Indian Institute of Technology, New Delhi, 110016 India; 2 Valetude Primus Healthcare Pvt Ltd, Okhla Phase 2, New Delhi, 110020 India; 3 National Institute of Pathology, Safdarjung Hospital, New Delhi, 110029 India; 4 Jamia Hamdard, Hamdard Nagar, New Delhi, 110062 India; 5 DRILS, University of Hyderabad Campus, Prof C.R. Rao Road, Hyderabad, 500046 India

**Keywords:** Tuberculosis, Laboratory techniques and procedures

## Abstract

Microscopy-based tuberculosis (TB) diagnosis i.e. Ziehl-Neelsen screening still remains the primary diagnostic method in resource poor and high TB burden countries, however this method has poor sensitivity (~60%). Bringing three million TB patients who are left undiagnosed under the treatment has been a major focus as part of END-TB strategy across the world. We have developed a portable set-up called ‘SeeTB’ that converts a bright-field microscope into fluorescence microscope (FM) with minimal interventions. SeeTB, a total internal reflection-based fluorescence excitation system allows visualization of auramine-O stained bacilli efficiently with high signal-to-noise ratio. Along with the device, we have developed a sputum-processing reagent called ‘CLR’ that homogenizes and digests the viscous polymer matrix of sputum. We have compared the performance of SeeTB system in 237 clinical sputum samples along with FM, GeneXpert and liquid culture. In comparison with culture as gold standard, FM has sensitivity of 63.77% and SeeTB has improved sensitivity to 76.06%. In comparison with GeneXpert, FM has sensitivity of 73.91% while SeeTB has improved sensitivity to 85.51%. However, there is no significant change in the specificity between FM and SeeTB system. In short, SeeTB system offers the most realistic option for improved TB case identification in resource-limited settings.

## Introduction

Tuberculosis (TB) is a major healthcare burden for developing countries, costing one human life every 20 seconds globally^1^. It is estimated that one in three of the world’s population is infected with *Mycobacterium tuberculosis* (*M.tb*), the causative agent of TB, but only 10% develop an active disease^2^. In 2017, 10 million people were suspected for TB and caused 1.6 million deaths^3^. TB can be cured if diagnosed early followed by complete course of treatment, currently only 58% of TB infections get diagnosed and treated^4^. In May 2014, World Health Organization (WHO) unanimously passed resolution to support ‘ENDTB strategy^5^. This strategy aims to reduce TB deaths by 95%, cut the new cases up to 90% by 2035, and complete eradication of TB by 2050. For eradication of TB, the number of cases must decrease at the rate of 16% per year^5^. Identification of three million undiagnosed patients is the major focus of END-TB strategy^5,6^.

TB detection was never thought as rapid diagnosis problem, now it is well established that early detection can limit the spread of new cases^7,8^. Given the fact that every unreported case of TB becomes the source of infection for 10–15 healthy persons^3^, for countries with high disease burden and poor resource availability it is imperative to adopt simple technology to ensure high case detection so as to interrupt the infection cycle. Hence, sputum smear microscopy, for the above reasons, will remain an important player in achieving the goals of WHO to eradicate TB.

India has the highest TB burden in the world, almost a quarter of TB cases happen in India with estimated economic loss of 40 billion USD^9^. India has been successful with TB treatment success rate of >85%, however new case detection rate is only 70%^10,11^. National Strategic Plan for TB control 11 envisions to detect 90% of TB cases by 2025. Sputum Smear Microscopy (SSM) continues to be a major method of TB diagnosis in primary health care settings. There are 14,000 designated microscopy centers in India and each caters to approximately 100,000 people in their proximity^12^. The new molecular based methods have shown promising sensitivity in TB detection^13–15^. The upgradation of existing TB diagnostic infrastructure is a costly affair and may not be possible in the next 10 years.

The SSM based method has been used for TB diagnosis for the past 100 years. SSM lacks sensitivity, only 50% of the cases are identified and takes 10 minutes hands-on time per sample^16^. Liquid culture still remains the gold standard, however culture of *M.tb* from sputum isolates takes 15 to 45 days^12^. The culture test requires biosafety containment (BSC) facility to grow the pathogens, which is a challenge in resource limited countries^17^. Recent advancements in Nucleic Acid Amplification Test (NAAT), has reduced the turn-around time and number of manual steps to improve the performance of TB diagnosis^13,18^. However, cost of large scale deployment of NAAT is a hurdle for low-middle income countries^8,19^.

Fluorescence microscopy (FM) of sputum smear slides for TB detection was proposed in 1930s and shown to improve the sensitivity as compared to the traditional Ziehl Neelsen (ZN) based test^4^. Sputum smears slides are stained using auramine-O dye, which specifically binds to mycolic acid in mycobacterium cell membrane^20^. The fluorescent stain lights up the cells in the dark background enabling easy identification of bacilli and reduces time taken for scanning per slide. Earlier FM set-ups used expensive halogen lamps and high compressed mercury lamps to illuminate the fluorophores that are expensive and needed replacement with time. The new Light Emitting Diode (LED) based FMs are cost effective and durable^4^. However limited sensitivity cost of device and reproducibility issues are still major barriers in large-scale adaption.

We have developed a novel portable set-up called ‘SeeTB’ that can convert a bright-field microscope into FM with minimal intervention. This compact total internal reflection fluorescence (cTIRF) based illumination device^21^ enables the fluorescence capture in brightfield microscopes. The SSM performance has shown large variability between operators due to the heterogeneous nature of the sputum sample. The sputum is a complex matrix with mucin molecules, actin filamentous molecules, cell debris, DNA etc. We have developed a novel sputum-processing reagent called CLR to breakdown the thick viscous sputum sample and digest it into a homogenous, almost transparent mixture. In this study, we have compared the performance of SeeTB system (comprising of SeeTB set-up with CLR reagent), in 237 clinical sputum samples along with FM, GeneXpert and liquid culture.

## Results

### SeeTB

**Figure 1:**
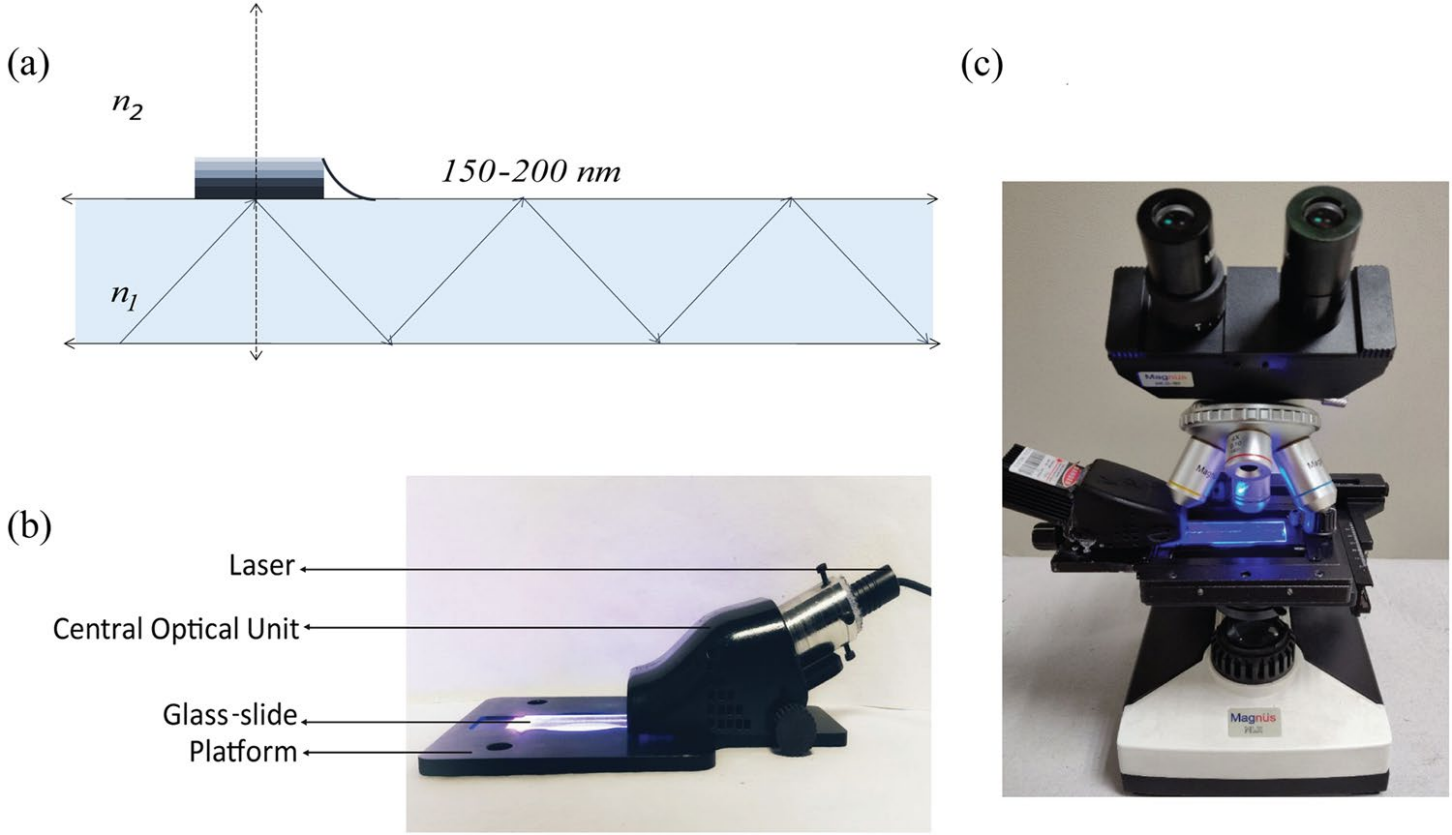
Schematic and actual SeeTB set-up. (**a**) Schematic of light wave-guide in flat glass slide (Cross section view). The incident light at an angle greater than critical angle gets totally internally reflected and propagates along the glass slide. At the glass-air interface, a small fraction of excitation light propagates to a maximum depth of 200 nm and selectively excites the fluorophore in that volume. (**b**) Compact TIRF excitation module ‘SeeTB set-up’. The metal platform with slots allows it to be mounted on most models of Bright-field microscope. All the optical components are assembled into a central optical unit (COU). Blue laser diode with 445 nm, is used as excitation source. (**c**) Brightfield microscope with a mounted SeeTB set-up.

Total internal reflection fluorescence microscopy (TIR-FM) has been extensively used for single molecule imaging of biological molecules^22^. When light moves from high refractive index (RI) medium to low RI medium at an angle greater than a critical angle^23^, the light gets totally internally reflected. At the interface of high-low RI boundary, a fraction of excitation light penetrates the low RI medium upto 150–200 nm called evanescent wave^21^ (Fig. 1a). Traditional FM involves Epi-mode of illumination; where the excitation light illuminates the whole cross-section in Z-axis of the sample under analysis. This results in exciting the fluorophores in whole volume of excitation beam and increases the background noise. In TIR, the excitation beam is highly selective to the fluorescent molecules close to the glass surface resulting minimum background from the sample. We developed a compact wave-guide based TIR excitation system (Fig. 1b), where light is coupled to a regular glass slide at critical angle to keep it totally internally reflected and generate evanescent wave throughout the surface of the glass slides^21^. This wave-guide based illumination has many unique advantages, as the excitation beam is perpendicular to the emission pathway, the number of optical components required to separate the excitation and emission light is minimal. Also the waveguide is generated with minimum number of pre-aligned optical components making it compact and affordable. This cTIRF system^21^ has been further modified to be used as an attachment to standard bright-field microscope (Fig. 1c) and upgrade any bright field microscope to TIR-FM.

**Figure 2:**
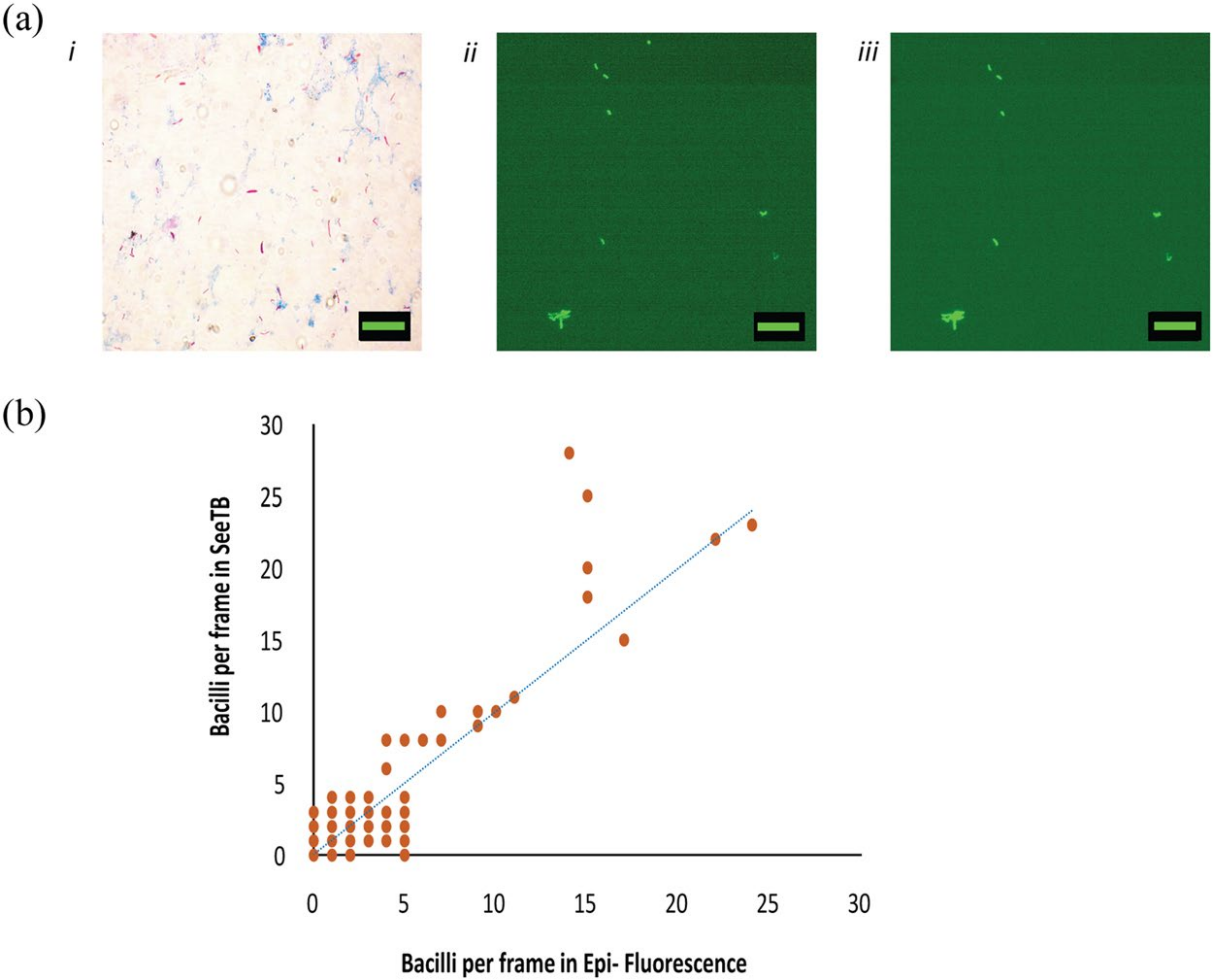
Comparative performance of SeeTB setup. (**a**) Left to right: (i) ZN stained sputum slide imaged using Brightfield M. (ii) Auramine-O stained slide imaged using FM. (iii) Image acquired using SeeTB set-up. (**b**) Comparison of number of bacilli counted in FM and SeeTB set-up.

Fluorescent microscopy of auramine-O stained sputum slides has many advantages over ZN stained SSM such as the ease of sample analysis i.e., identification of bacilli is efficient and faster^4^. In ZN staining, to differentiate a bacilli shape from the background, such as the high magnification 100X oil objective is used^24^. In FM, auramine-O staining is highly specific to mycolic acid in the membrane of mycobacterium species (Fig. 2a). Binding of these fluorescent dyes lights up the cell surface against dark background enabling efficient and rapid identification of bacilli. Auramine-O stained sputum smear slides were imaged using both standard epi-excitation in FM and in TIR-FM mode using SeeTB system. The major difference between the FM and TIR-FM is volume of sample illuminated, in SeeTB system the penetration depth of evanescent wave is about 160 nm^21^. Though the volume of illumination is lower in comparison to EPI-illumination, 99% of cells could be easily identified using SeeTB illumination (Fig. 2b). Shape of the mycobacterium cells could be readily identified using SeeTB system with enhanced signal-to-noise ratio (SNR) in comparison to FM (Supplementary Fig. S1). Only cells that are close to the glass surface light up. Due to restricted nature of excitation beam profile the SeeTB illumination had better discriminatory power to avoid background noise from larger debris in sputum.

**Figure 3:**
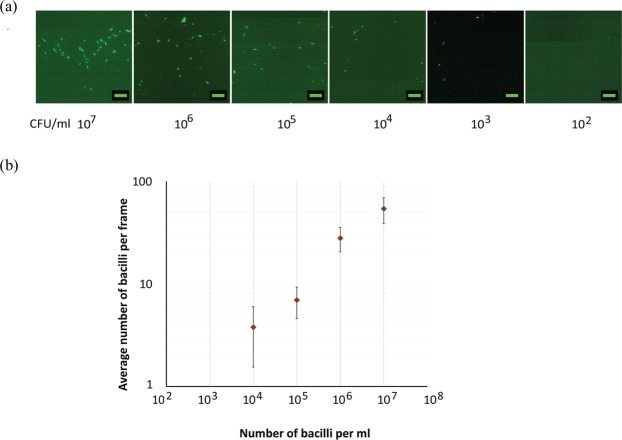
Limit of detection in SeeTB system (SeeTB setup + CLR). (**a**) *Mycobacterium tuberculosis* H37Ra cells stained using auramine-O. Cells were serially diluted and images acquired at randomly in 30 frames. (**b**) Average number of bacilli visualized using SeeTB set-up.

To further test the performance of SeeTB set-up*, Mycobacterium tuberculosis* H37Ra cultured cells (concentration 10^8^ per mL) were used to prepare different dilutions. These serially diluted samples were stained using auramine-O and enumerated using SeeTB (Fig. 3a). Number of bacilli per frame was countable upto 10^4^ bacilli/mL (Fig. 3b). By increasing the number of fields scanning to 10 frames, the limits of detection (LOD) was further enhanced to 1000 bacilli/mL.

**Figure 4:**
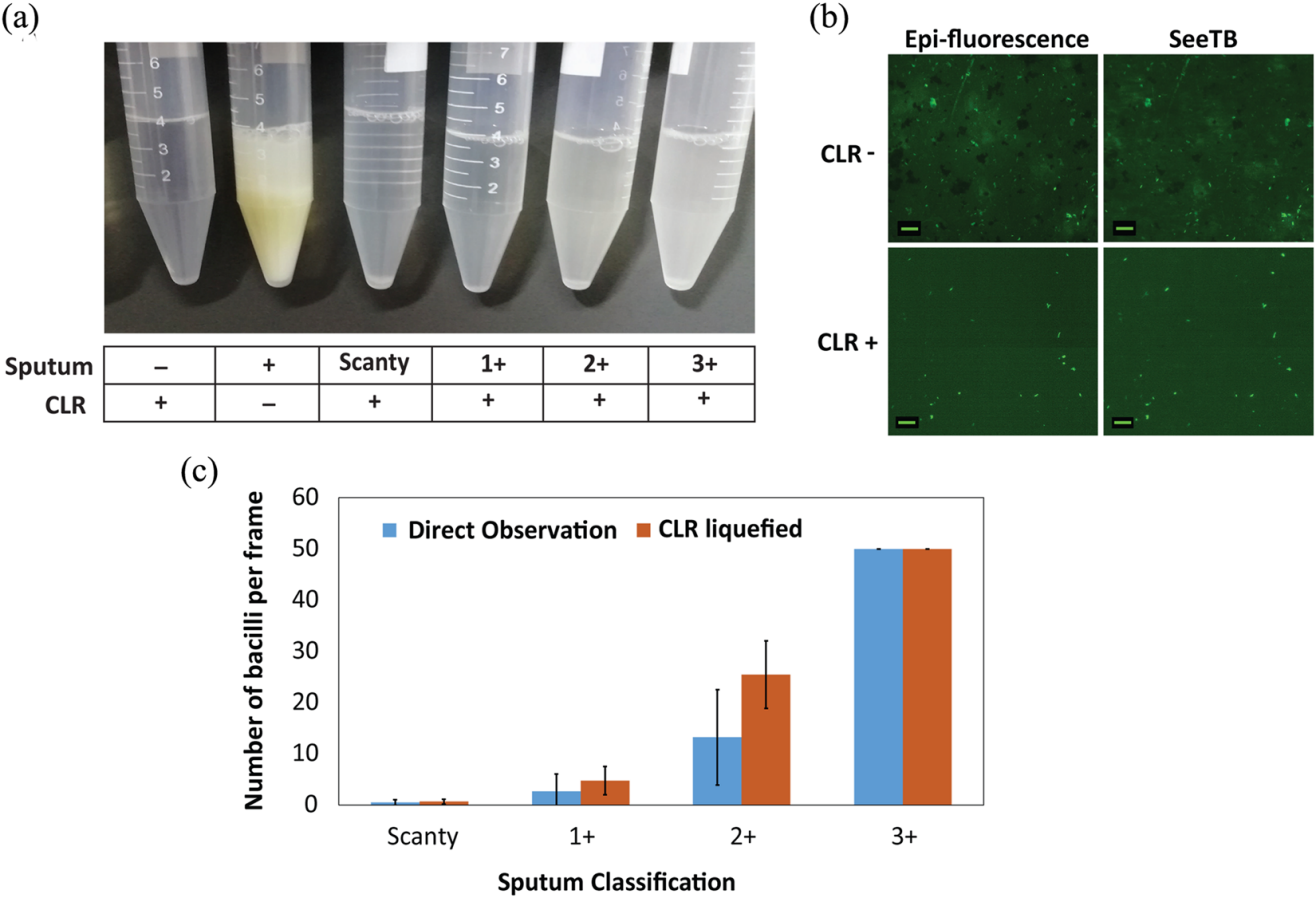
Comparative performance enhancement of CLR. (**a**) Comparison of optical clarity of sputum samples with and without CLR treatment. All different grades of sputum samples are rapidly homogenized with CLR treatment. (**b)** Comparison of background staining of auramine-O with and without treatment of CLR reagent. (**c)** Average number of bacilli enumerated with (red bar) and without CLR treatment (blue bar).

Auramine-O sputum smear imaging still had many disadvantages i.e., sample that is used for microscopy is non-homogenous. When a highly viscous sputum sample is handled, a small toothpick is used to pick a loopful of sputum. This random sampling has introduced high variability between different operators and decreasing the reproducibility of the assay. We have developed a novel sputum processing reagent called ‘CLR’, comprising of DNAase, trypsin, sodium dodecyl sulfate (SDS) and dithiothreitol (DTT) that effectively depolymerizes the networked mesh of sputum. This rapid thinning process reduced sputum viscosity, enhanced sample homogeneity and released the clustered Acid Fast Bacilli (AFB) in the solution. CLR worked efficiently at 37 °C and within 15 minutes of reagent addition, the sputum samples liquefied into transparent solution. Even the most viscous sputum samples get dissolved into homogenous transparent solution. The performance of the thinning reagent, unprocessed sputum along with CLR processed sputum is shown in Fig. 4a. All the macroscopic objects in the unprocessed sputum increase the non-specific absorption of auramine-O dye and lower the efficiency of the de-staining step. With CLR treatment, the background noise is significantly reduced enabling easy identification of AFB (Fig. 4b). Evaluation of the number of bacilli with and without CLR reagents in positive/negative TB patients showed clear increase in the total number of AFB identified. Number of AFB per frame increased by ~40% for scanty, 1+ and 2+ classified sputum samples. There was no measurable difference in 3+ sputum samples before and after CLR treatment (Fig. 4c).

**Figure 5:**
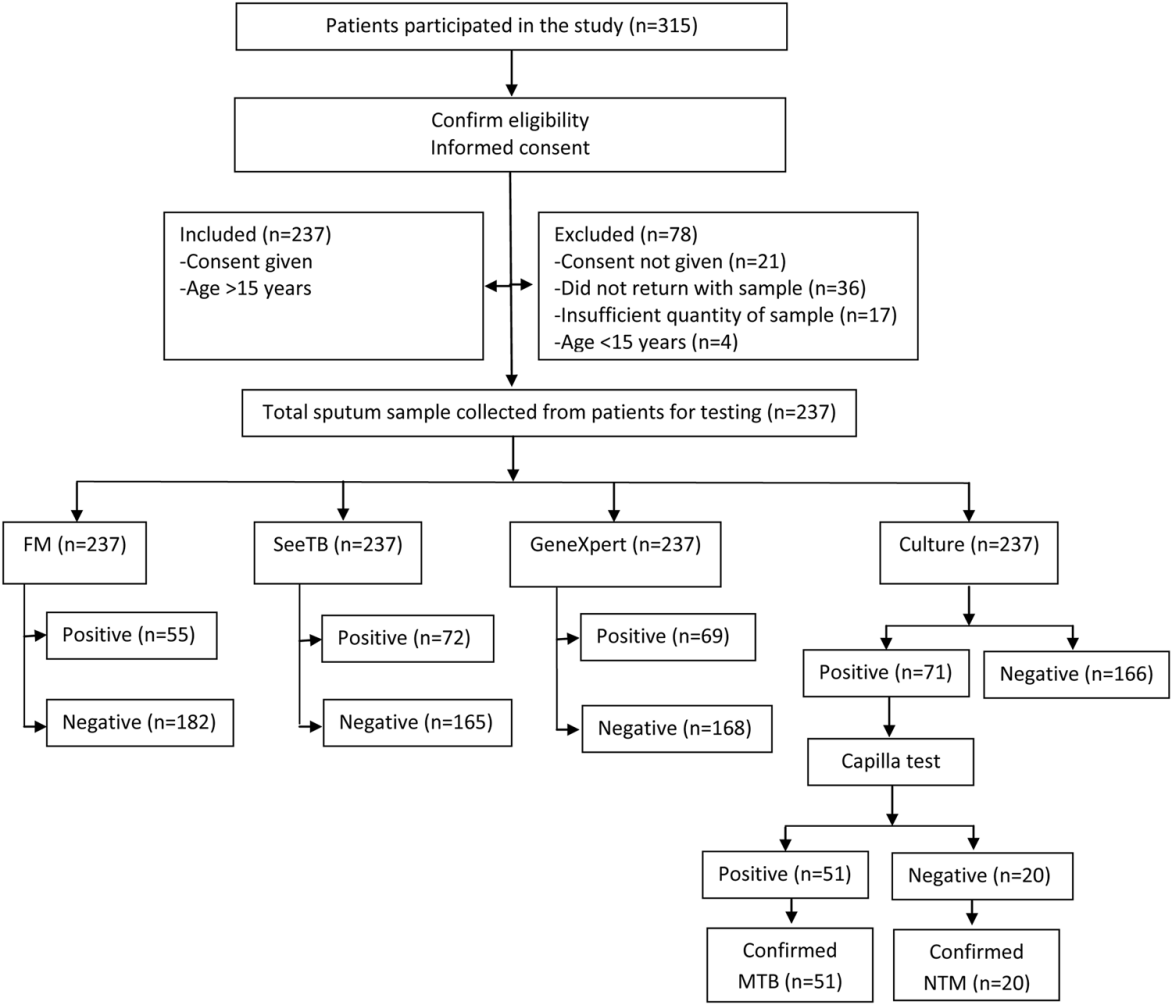
Flow of participants in the study.

The present study enrolled 315 clinically suspected pulmonary TB patients. Of 315 patients 237 patients samples were included in the study and 78 were excluded from the study due to insufficient quantity of sputum, absence of consent or insufficient comparative test results. The flow of patients participated in the study is summarized in Fig. 5. The median age of the patients was 35 years, 130 (54.9%) were women and 107 (45.1%) were men. Of 237 samples, 71 (30%) cases were positive and 166 (70.8%) were negative by culture, 69 (29.1%) were positive and 168 (70.8%) were negative by GeneXpert. It is important to note that of the 71 positive cases by culture, 51 were *M.tb* and 20 were Non Tuberculous Mycobacteria (NTM). Of 237 samples, 55 (23.2%) cases were positive and 182 (76.7%) were negative by FM, 72 (26.2%) were positive and 165 (69.6%) were negative by SeeTB.

In direct comparison with FM, SeeTB detected 22 (9.3%) more positive cases declared as negative by FM and missed 5 (2.1%) cases declared as positive cases by FM (Table 1). Of these 22 cases, 11 were positive by GeneXpert and 6 NTM cases by culture. There were 5 negative cases by SeeTB but identified as positive in the FM. These 5 cases were compared with the GeneXpert and culture results. While 1 sample was found positive by GeneXpert and negative by the culture however another 1 case that was detected positive by GeneXpert, was found to be NTM by the culture test. And rest 3 cases were culture negative (Table 1).

**Table 1:** Comparative performance analysis of FM and SeeTB against GeneXpert and Liquid Culture.

FM	SeeTB	Total sample	GeneXpert		Liquid Culture		
			Positive	Negative	MTB	NTM	Negative
Positive	Negative	5 (2.1%)	3	2	1	1	3
Positive	Positive	50 (21.1%)	48	2	43	0	7
Negative	Positive	22 (9.3%)	11	11	5	6	11
Negative	Negative	160 (67.5%)	7	153	2	13	145
Total		237	69	168	51	20	166

Taking culture as gold standard, we compared the performance of FM and SeeTB system. Against culture, FM showed 63.38% sensitivity and 93.97% specificity. SeeTB system showed improved sensitivity of 76.05% and minor reduction in specificity to 89.15%. When SeeTB system and FM performance was compared against GeneXpert, FM showed 73.91% sensitivity and 97.61% specificity, however, SeeTB system showed improved sensitivity of 85.50% and minor reduction in specificity to 92.26%. The sensitivity of the SeeTB for detection of TB in sputum sample was statistically significant than that for FM (*p* = < 0.0001).

In comparison with culture, 18 additional cases were found positive by SeeTB that were culture negative. Of 18 positives cases by SeeTB system, comparing along with GeneXpert results, we found 13 samples were GeneXpert positive and only 5 false positive cases. In comparison with culture, 17 cases were missed by SeeTB while the same showed culture growth, 14 were NTM and 3 were TB cases. Excluding the NTM cases, overall SeeTB system had 5 false positive and 3 false negative cases reflecting 95.71% sensitivity and 97.01%. The positive predictive value (PPV) of 93.1% while negative predictive value (NPV) was 98.2% of SeeTB system (Table 2).

**Table 2:** Sensitivity and specificity of SeeTB and FM against culture and GeneXpert.

	Comparison with Culture		Comparison with Xpert	
	FM	SeeTB	FM	SeeTB
False Negative	26	17	18	10
False Positive	10	18	4	13
True Negative	156	148	164	155
True Positive	45	54	51	59
Sensitivity	63.38%	76.05%	73.91%	85.50%
Specificity	93.97%	89.15%	97.61%	92.26%

## Discussion

### Advantages of SeeTB

The SeeTB set-up, based on cTIRF, can be added to the bright-field microscope to upgrade it to FM. The system is designed with a robust metal platform and all the optical components are prealigned in a plastic casing. The use of the SeeTB set-up is straight forward, the central optical unit can be moved to add a new slide. To our knowledge, this is the only TIR imaging system applied for TB diagnosis. This perpendicular illumination system reduced the number of optical components required for separating excitation and emission beam. SeeTB system with 160 nm illumination depth is enough (Figs 2b and S1) to clearly identify the shape of the bacilli. TIR based illumination reduces the background noise and enhances the identification of bacilli. There are many instances where the number of bacilli is more in SeeTB system than the conventional FM. As overlapping cells and debris that non-specifically bind to fluorescent dyes can mask the shape of cell, but in TIR-FM additional Z axis selection gives more specificity to the discrimination of cell shape.

### Advantages of CLR reagent

CLR reagent used in this study enhances the performance of the SeeTB system. The sputum is a complex, heterogeneous sample with lot of protein and DNA polymers entangling the mixture in macroscopic suspension^25^. Due to heterogeneous nature of sample there is performance variation across different operators as the sample picked is seldom the same composition. Another disadvantage of FM is that mycobacterial cells are masked by the polymeric mesh which does not allow the penetration of auramine-O dye. CLR reagent has been specifically designed to depolymerize the protein and DNA mesh to enhance the solubility of sputum components in the solutions in minimum duration. The CLR reagent completely homogenizes the sputum sample within 15 minutes and reduces the viscosity of the solution almost equivalent to the CLR solution. This homogenization and reduction of particulate matter enhances the release of bacilli in solution and increases the efficiency of fluorescent dye labeling. We have used 40 μL of liquefied sputum to prepare auramine-O stained smear slide. WHO recommended protocol only requires 10 μL of sputum for preparing the smear. In our case, since the sample is liquefied we are able to use 40 μL of the liquefied sputum sample and heat fix it to prepare the auramine-O stained slides. This modification did not affect the intensity of bacilli staining, while the use of CLR reagent enabled reduction of non-specific labeling in the slides. This modified protocol of 40 μL smear slides enhances the LOD of SeeTB system to 10^4^ bacilli per mL. Based on the SeeTB and CLR reagent performance; we used 30 fields scanning along with CLR liquefaction of sputum in clinical samples. SeeTB, in comparison with FM, showed improvement of 10–15% sensitivity and equivalent specificity. Due to improved sensitivity, SeeTB system could detect 30% more positive cases over FM.

Though culture diagnostics is considered as gold standard there are certain drawbacks in comparison to molecular diagnostic assays. The positive results in the culture depend on the quality of the sputum preparation i.e. what method of sputum decontamination is used and if there are any enrichment methods like centrifugation is used in the process. Also, culture result varies if the patient is under Anti-Tubercular Treatment (ATT)^11^. Using SeeTB we identified 18 (7.5%) additional cases that were missed in the culture test. Of all culture negative and SeeTB positive cases, 70% were found positive in GeneXpert. This means, there were *M.tb* present in the sample, but not viable in culture medium.

Liquid culture identifies not only *M.tb*, but also NTM in sputum sample. GeneXpert being a nucleotide based diagnostics, it is highly specific for *M.tb* species and NTM cases are not identified in GeneXpert. However, if we only consider *M.tb* cases, there were only 3 true cases missed and only 5 false positive cases reported. These imply a PPV and NPV of 93.1% and 98.2%, respectively. Looking at the very high NPV, we see a strong case of SeeTB system as screening test for identifying patients who need further detailed characterization. We plan to further extend this study in multi-center mode and work on the stability of CLR reagent for effective transportation and storage.

In our quest to upgrade the existing diagnostic infrastructure to highly sensitive system efforts have been focused so far only on evaluating nucleotide amplification test (NAAT). Upgrading the diagnostic infrastructure must also address issues of device cost, point-of-care infrastructure availability, requirement of trained manpower and rapid turn-around time. For large scale screening to maximize case identification, the cost per test is also an important variable. Among the many molecular assay methods, cost per test is lowest for auramine-O smear microscopy, but poor sensitivity has been the challenge. Based on the raw material cost, we estimate ~700 USD could be the commercially sustainable pricing for our SeeTB. A comparison of many existing fluorescence microscopy systems available (Table 3) reveals that SeeTB is a cost effective solution for upgrading brightfield microscope to FM. It has not escaped our attention that the device SeeTB, based on total internal reflection, will have other applications, including use in cytology & pathology labs where fluorescent microscopes are frequently used to detect tumor samples and other infections.

**Table 3:** Comparison of SeeTB system with other existing fluorescence imaging technologies.

Technology	Type	Cost per device
LED-FM (Carl Zeiss AG)	Epi-fluorescence (Transmission mode)	$1700
SeeTB + CLR (Valetude Primus Healthcare Pvt. Ltd.)	Evanescent wave based illumination (Perpendicular mode)	$700
FluoLED (Fraen Corporation)	Epi-fluorescence (Transmission mode)	$1500
Lumin (LW Scientific Accessories)	Epi-fluorescence (Transmission mode)	$1758–$ 2500

## Conclusion

The CLR-SeeTB system has shown improved sensitivity and equivalent specificity compared to conventional FM for the detection of AFB in patient samples. While stability of CLR at ambient temperature still needs to be worked out, the advantages of the SeeTB system are sensitivity, high negative predictive value, affordability, deployability and scalability. In short, our system offers the most realistic option for improved TB case identification in high disease burden and resource-limited settings. Large-scale field trials are required to assess the advantages and feasibility of replacing traditional FM with SeeTB as a first-line diagnostic test.

## Materials and Methods

### Ethics approval and study setting

Clinical sputum samples were collected from two hospital sites; Safdarjang Hospital, Delhi and DOTS TB Clinic at the Jamia Hamdard Hospital, Delhi. All the protocols of this study were approved by the Institute Ethics Committee in Safdarjung Hospital and Jamia Hamdard Hospital and the methods were performed in accordance with the Revised National TB Control Programme (RNTCP) guidelines and regulations. Suspected TB patients who were able to produce mucoid/purulent sputum (~5 mL) and above 15 years of age were included in the study. Patients unable to produce adequate amount of sputum sample and HIV positive cases were excluded from the study. The informed consent from patients willing to participate has been taken from parent and/or legal guardian for study participation.

The patients with clinical suspicion of pulmonary tuberculosis including symptoms of cough for more than 2 weeks, weight loss, hemoptysis, mild fever and loss of appetite were recommended by the physician for the study. The essential identification information of the patients such as name, age and gender were recorded by trained personnel. The sputum samples were collected in sterile and leak-proof containers. A unique number was assigned to each sample considered for the study. The sputum samples collected on first day and on next day morning were pooled together for evaluation through GeneXpert, Liquid Culture, FM and SeeTB test. All laboratory tests were performed using standard operational procedures. The BSC was used during sample preparation including initial homogenization and sample splitting, culture inoculation and drug susceptibility test setting to ensure safety and avoid risk of contamination. For clinical performance study, the samples were kept blinded to the SeeTB operator i.e. without any prior knowledge of the other test results. The data from clinical tests and SeeTB test were sent to a third person for record keeping and analysis.

### Mycobacterium culture on liquid medium

As the samples arrived at designated laboratory site, a part of the sputum sample (~1 mL) was decontaminated using N-acetyl-L-cysteine (NALC)-sodium hydroxide NaOH (4%). After concentration by centrifugation at 3000 g for 15 min, the sediment was re-suspended with 2 mL of 0.5 M phosphate buffer (pH 6.8) and inoculated in Middle Brook 7H9 broth of BacT/ALERT MP process bottle (BioMerieux) and incubated at 37 °C for 7 weeks. Each positive culture was inoculated on blood agar media and incubated for 48 h to rule out any contamination. The isolate from the culture bottle was confirmed as acid fast bacilli by ZN staining and distinguished as Mycobacterium TB Complex (MTBC) or Non Tuberculosis Mycobacterium (NTM) using the TB Ag MPT64 Rapid Test (SD Bioline, Kyonggi-do, Korea). All procedures were carried out inside a BSC by using full personal protective equipment.

### GeneXpert MTB/RIF (GeneXpert) assay

Sputum samples were treated with sample reagent (SR) containing NaOH and isopropanol provided as per the manufacturer’s instruction (Cepheid manual, 2012)^26^. The SR was added using a 2:1 ratio of the sputum sample, homogenized and incubated for 15 min at room temperature. From the treated samples 2 mL was transferred into multi-chambered plastic cartridge preloaded with liquid buffers and lyophilized reagent beads necessary for sample processing, deoxyribonucleic acid (DNA) extraction and hemi-nested real-time polymerase chain reaction (RT-PCR). The cartridge was loaded into the GeneXpert machine (GeneXpertRDx System version 4.8, Cepheid, Sunnyvale, CA USA), and an automatic process completed the remaining assay steps. The results were visualized in printable form in the view results window.

### Fluorescence microscopy

The FM was conducted on the conventional LED-FM microscope (Carl Ziess Primo Star iLED) in accordance with the International Union Against Tuberculosis and Lung Disease working group on smear microscopy^20^. All the direct smear and auramine-O stained slides were examined at 200x LED-FM (Carl Ziess Primo Star iLED) with confirmation of positive smears at 400 × magnification. The number of AFB read per standard length of 2 cm long was reported. A length corresponding to 100 fields under 1000x magnifications was estimated to be equivalent to 20 fields under 400x magnifications. The cost of SeeTB system attachment was compared with existing attachments consisting of a specialized objective with an attached LED light source (Lumin and the QBC ParaLens Fluorescence Microscopy System).

### SeeTB set-up

The SeeTB set-up was designed to generate the large area evanescent waves in the regular glass slides. Device has two major components: First, the base metal sheet with grooves for mounting the device on the microscope and grooves within the base sheet for the mounting the glass slides on the device; Second, the central optics unit with light shaping optical components. All the optical components are pre-assembled and arranged into compact central optical unit. All the optical components were mounted with precise alignment on a plastic casing. The SeeTB device uses right-angled prism for coupling the light (blue 445 nm, 100 mW, from Berlin Optics, Germany) into the flat glass substrate (75 mm × 25 mm × 1 mm). A cylindrical lens is used to focus the laser beam on the prism, which later couples the light into the glass substrate. For new slide, a small drop of immersion oil was added between the glass slide and central optical unit to enable reliable optical coupling. Central optical unit can be tilted up to 40 degree to easily add or remove a glass slide. For each new slide, a drop of immersion oil is added to couple the light between central optics unit and glass slide. Blue laser with 445 nm emission was used as excitation light source. Emission was selected using band pass filter added to eyepiece. The bright-field microscope can be upgraded to TIR fluorescence system with SeeTB set-up with minimal intervention. The emission light was filtered using a 6 band-pass filter (Kodak wratten filter, yellow 12, #54–467, Edmond optics), added to both the eyepiece. This linear waveguide generated large area evanescent waves on upper and lower surface of the glass substrate.

### Composition of CLR reagent

20 mL stock solution of 10X CLR was prepared for use in this study with following composition; trypsin (0.25 g), DTT (0.30 g), 10% SDS (4 mL), DNase 4000 U/mL (0.5 mL), Sodium Citrate (3 g), nuclease free water (15.5 mL). Stock solution was kept in −20 degree until use.

### SeeTB protocol

**Figure 6:**
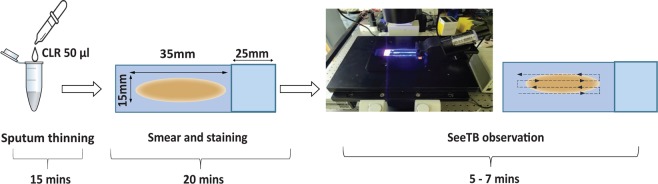
Schematic of clinical protocol of the study.

SeeTB microscopy was conducted on bright field microscope (Bright-field microscope) attached with SeeTB set-up on the platform. For every clinical sample, 50 μL of sputum was taken into an eppendorf tube, 50 μL of 4x CLR reagent was added, thoroughly vortexed and incubated for 15 mins for complete thinning. The 40 μL of completely homogenous mixture was spread on a slide. An area of approximately 35 mm × 15 mm was used for smear preparation. The sputum was spread to make a smear using circular movements and allowed to air dry. The heat fixed slides were placed on the staining rack carefully keeping the smear part facing up. The slides were stained with auramine-O as per the WHO recommended fluorescent staining protocol^25^. A length equivalent to 30 fields under 400x magnifications was used to scan the sputum sample to evaluate the performance. An experienced lab scientist reported the results using SeeTB set-up. The clinical protocol of the study is shown in the Figs 6 and S2.

### Limit of detection (LOD) using SeeTB set-up

The MTB strain H37Ra was used to estimate the LOD using SeeTB set-up. The MTB H37Ra cells were grown on Middlebrook 7H9 broth (Difco Laboratories) and 0.05% Tween 80 (Sigma-Aldrich) enriched with oleic acid-albumin-dextrose-catalase (Difco Laboratories) at 37 °C with continuous agitation. The grown cells were washed with 1X PBS by centrifugation at 3500 rpm for 10 min, finally suspended in 1X PBS to O.D_600_ of 0.8. The bacterial suspensions were serially diluted in 1: 10 dilution from 10^7^ to 10^2^ CFU/mL. The slide was cleaned and labeled by the diamond marker. The smear of each dilution was prepared in front of the flame and subsequently heat-fixed. The heat-fixed slides were stained with acid-fast staining with auramine-O. The staining of smear was done as per the WHO recommended protocol^24^. Visibly stained bacilli were counted in 30 frames at 400X magnification, viewing at least 30 fields, along with positive and negative control slides. We evaluated the LOD by using bright-field microscope in conjunction with SeeTB set-up.

### Digital image acquisition and statistical analysis

Comparative FM and SeeTB images were taken by mounting SeeTB device on Olympus IX71 FM with Andor Xyla sCMOS camera with 50X long working distance objective. The acquired images were stored as TIFF files and analyzed using ImageJ (https://imagej.nih.gov/ij/) open access software. The data were tabulated in the Microsoft ExcelR Spreadsheet in the master chart and studied for the correlation. A statistical macro-code was written to analyze data in Excel sheet. The sensitivity, specificity, PPV and NPV were calculated with respect to GeneXpert, and liquid culture.

## Supplementary information

SeeTB: A novel alternative to sputum smear microscopy to diagnose tuberculosis in high burden countries

## References

[1] Zumla A (2015). The WHO 2014 Global tuberculosis report—further to go. Lancet Glob. Heal..

[2] Milburn H (2007). Key issues in the diagnosis and management of tuberculosis. J. R. Soc. Med..

[3] Global Tuberculosis Report 2018. (2018).

[4] Steingart KR (2006). Fluorescence versus conventional sputum smear microscopy for tuberculosis: a systematic review. Lancet Infect Dis..

[5] Uplekar M (2015). WHO’s new End TB Strategy. Lancet.

[6] Herbert N (2018). Advancing political will to end the tuberculosis epidemic. Lancet Infect Dis.

[7] Mandal S, Arinaminpathy N (2015). Transmission modeling and health systems: the case of TB in India. Int. Health.

[8] Arinaminpathy N, Dowdy D (2015). Understanding the incremental value of novel diagnostic tests for tuberculosis. Nature.

[9] Kirigia JM, Muthuri RDK (2016). Productivity losses associated with tuberculosis deaths in the World Health Organization African region. Infect. Dis. poverty.

[10] Revised National TB Control Programme Annual Status Report (2018).

[11] Sachdeva KS, Kumar A, Dewan P, Kumar A, Satyanarayana S (2012). New vision for Revised National Tuberculosis Control Programme (RNTCP): Universal access - &quot;reaching the un-reached&quot. Indian J. Med. Res..

[12] Revised National TB Control Programme Training Manual for *Mycobacterium tuberculosis* (2009).

[13] Nikam C (2013). Rapid Diagnosis of Mycobacterium tuberculosis with Truenat MTB: A Near-Care Approach. PLoS One.

[14] Rachow A (2011). Rapid and Accurate Detection of *Mycobacterium tuberculosis* in Sputum Samples by Cepheid Xpert MTB/RIF Assay-A Clinical Validation Study. PloS One.

[15] Global Tuberculosis Programme. The use of loop-mediated isothermal amplification (TB-LAMP) for the diagnosis of pulmonary tuberculosis: policy guidance (2016). 27606385

[16] Alnour TMS (2018). Smear microscopy as a diagnostic tool of tuberculosis: Review of smear negative cases, frequency, risk factors, and prevention criteria. Indian J. Tuberc..

[17] Shi J (2018). GeneXpert MTB/RIF Outperforms Mycobacterial Culture in Detecting *Mycobacterium tuberculosis* from Salivary Sputum. Biomed Res. Int..

[18] Zeka AN, Tasbakan S, Cavusoglu C (2011). Evaluation of the GeneXpert MTB/RIF Assay for Rapid Diagnosis of Tuberculosis and Detection of Rifampin Resistance in Pulmonary and Extrapulmonary Specimens. J. Clin. Microbiol..

[19] Pantoja A, Kik SV, Denkinger CM (2015). Costs of Novel Tuberculosis Diagnostics—Will Countries Be Able to Afford It?. J. Infect. Dis..

[20] Cuevas LE (2011). LED Fluorescence Microscopy for the Diagnosis of Pulmonary Tuberculosis: A Multi-Country Cross-Sectional Evaluation. PLoS Med..

[21] Pandey V, Gupta S, Elangovan R (2017). Compact 3D printed module for fluorescent and label-free imaging using evanescent excitation. Methods Appl. Fluoresc..

[22] Axelrod D (2001). Total internal reflection fluorescence microscopy in cell biology. Traffic.

[23] Mattheyses AL, Simon SM, Rappoport JZ (2010). Imaging with total internal reflection fluorescence microscopy for the cell biologist. J. Cell Sci..

[24] Laboratory Diagnosis of Tuberculosis by Sputum Microscopy The handbook Global edition A publication of the Global Laboratory Initiative a Working Group of the StopTB Partnership global laboratory initiative advancing TB diagnosis (2013).

[25] Akhtar, M. *et al*. Technical Guide: Sputum Examination for Tuberculosis by Direct Microscopy in Low Income Countries. IUATLD Fifth Ed (2000).

[26] Genexpert Xpert Mtb Rif User Manual Pdf Download. Available at, https://www.manualslib.com/manual/1197251/Genexpert-Xpert-Mtb-Rif.html (Accessed: 8th November 2018) (2012).

