# Supplementary material for: SeeTB: A novel alternative to sputum smear microscopy to diagnose tuberculosis in high burden countries
Source: Sci Rep. 2019 Nov 12;9:16371. doi: 10.1038/s41598-019-52739-9 (PMC6851358; doi:10.1038/s41598-019-52739-9)
Supplement: Supplementary file 1 — SeeTB: A novel alternative to sputum smear microscopy to diagnose tuberculosis in high burden countries [file 41598_2019_52739_MOESM1_ESM.pdf]

**Title: SeeTB: A novel alternative to sputum smear microscopy to diagnose tuberculosis in high burden countries.**

**Authors:** Vikas Pandey[1,2,†], Pooja Singh[1,†], Saumya Singh[1], Naresh Arora[1], Neha Quadir[3], Saurabh Singh[1,2], Ayan Das[4], Mridu Dudeja[4], Prem Kapoor[4], Nasreen Zafar Ehtesham[3], Ravikrishnan Elangovan[1,2], and Seyed E. Hasnain[1,2,4,5,*]

## Supplementary figures

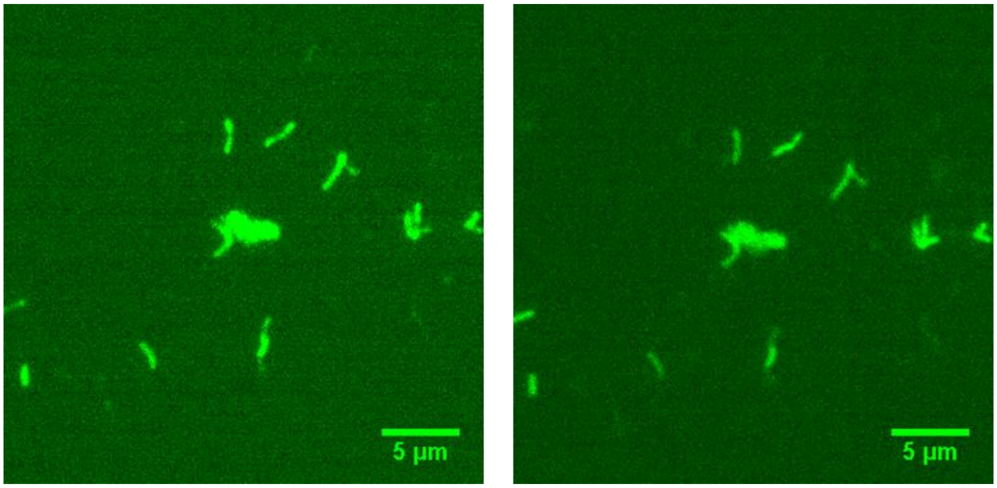


**Fig. S1. Comparative images of magnified bacilli using SeeTB system (right) and Epi-fluorescence (left)**

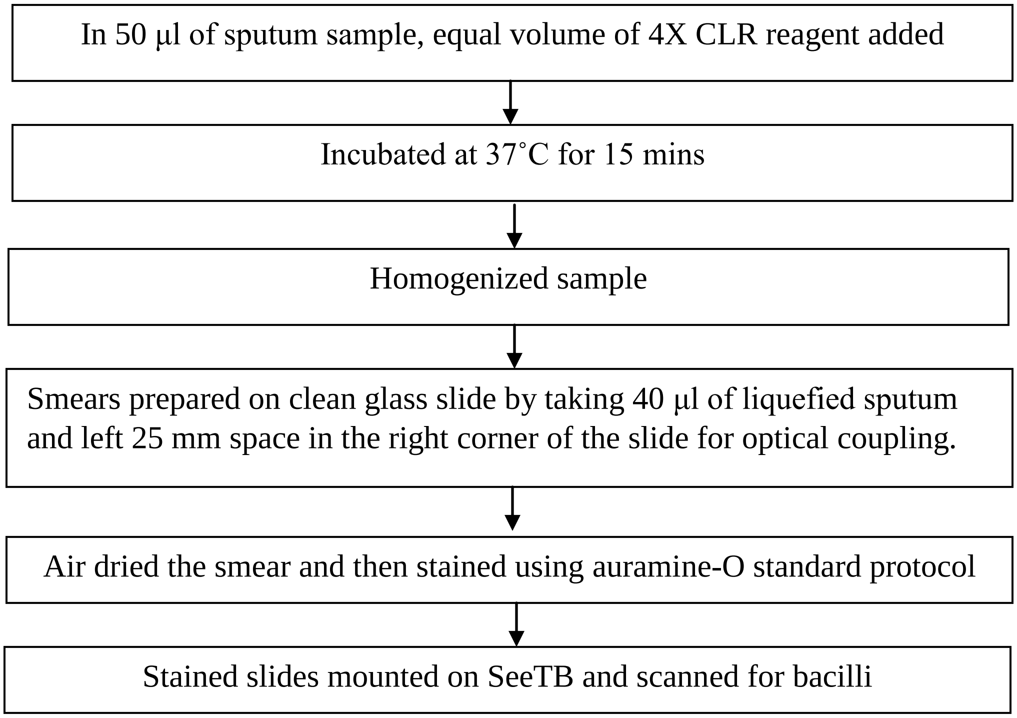


**Fig. S2: Clinical protocol of the study**
